# Proximal Femur Fractures: Evaluating the Necessity of On-Call Surgery

**DOI:** 10.3390/jcm14010093

**Published:** 2024-12-27

**Authors:** Vanessa Ketter, Antonius Korschinsky, Ulf Bökeler, Rene Aigner, Benjamin Bücking, Daphne Asimenia Eschbach, Katherine Rascher, Steffen Ruchholtz, Tom Knauf

**Affiliations:** 1 Center for Orthopaedics and Trauma Surgery, University Hospital Giessen and Marburg GmbH, 35043 Marburg, Germanysteffen.ruchholtz@med.uni-marburg.de (S.R.); 2 Departement 20 Human Medicine, Philipps-University Marburg, 35037 Marburg, Germany; 3 Marienhospital Stuttgart, 70199 Stuttgart, Germany; 4 Helios Kliniken Kassel, 34121 Kassel, Germany; 5 MVZ Hessisch Lichtenau e.v., Kaufungen and Kassel, 34123 Kassel, Germany; 6 AUC—Akademie der Unfallchirurgie GmbH, 80538 Munich, Germany

**Keywords:** geriatric, nightshift surgery, proximal femur fractures, geriatric, time to surgery

## Abstract

**Background:** The decision of the Joint Federal Committee on the treatment of hip fractures stipulates that proximal femur fractures must be treated within the first 24 h. This leads to organizational and personnel difficulties in day-to-day care. Therefore, we investigated the question at what times of day we operate to maintain this timeline and whether there is a difference in the outcome for the patients according to treatment hours. **Methods:** Data from the DGU’s “AltersTraumaRegister” from 2016 to 2020 were analyzed. For the analysis, the patients were divided into seven cohorts depending on the time of surgery. Pre-operative, operative, and follow-up data were analyzed. **Results:** A total of 29,470 patients were included in our study. The results showed that 74% of patients were treated within 24 h. 72% of patients operated on between 0–7 h had pertrochanteric fractures, while 56% of all arthroplasties were performed during normal working hours. In supra-regional trauma centers, significantly fewer operations were performed during normal working hours, while significantly more surgeries were carried out in the late evening and at night (*p* < 0.001). There were no significant differences in mortality and morbidity between the individual groups. **Conclusions:** Although we manage to treat most patients within 24 h, only 46% of patients are operated on within normal working hours. In terms of the outcome parameters, this does not appear to be a disadvantage for the patients. Nevertheless, night work and fatigue affect concentration and post-operative results in many areas, as we know. Consequently, patient care during normal working hours within 24 h creates the best possible initial situation for the patient, as significantly more personnel resources are available during normal working hours. The aim should be to create the logistical and personnel requirements for this.

## 1. Introduction

Proximal femoral fractures are considered typical fractures in geriatric patients [1]. According to numerous studies, the number of these fractures will increase significantly in the future [2]. The treatment of geriatric trauma patients presents structural, personal, and economic challenges for healthcare providers [2]. Despite the effort involved in introducing orthogeriatric co-management and the aim of optimizing surgical care and long-term rehabilitation measures, the treatment of patients with proximal femur fractures remains complicated. The post-operative course is associated with a high degree of permanent morbidity, reduced quality of life, high mortality rate, and limited mobility and independence [3,4].

The ideal timing of surgery for patients with proximal femoral fractures is under discussion. In general, patients should receive surgical treatment as soon as possible [5]. There is mainly consensus that prompt treatment, within 24 h, is the best possible treatment for most patients, leading to a significantly lower mortality [6]. However, an analysis of data from the geriatric trauma registry shows that it makes no difference—at least for patients who are cared for in an orthogeriatric setting—whether they are treated within 24 or 48 h [7].

Irrespective of all discussions about the time of surgery, the Joint Federal Committee on the treatment of proximal femoral fractures determined in 2021 that these fractures must be treated promptly within the first 24 h [8]. It is possible that this will lead to organizational difficulties in everyday care due to current resources and staff shortages. The question, therefore, arises as to whether it is realistic to adhere to this treatment timeline and at what time during working hours should these fractures be treated. We asked ourselves whether treatment late in the evening or at night results in a difference in the outcome for these patients.

## 2. Materials and Methods

We extracted data from the “AltersTraumaRegister DGU©”, a prospectively collected registry of geriatric trauma patients undergoing surgery for hip-related fracture or periprosthetic fracture in an orthogeriatric setting.

Hospitals from Germany, Austria and Switzerland are currently participating in the “AltersTraumaRegister DGU©” with about 16,000 cases from 150 hospitals per year.

For every certified “Centre for Geriatric Trauma” (AltersTraumaZentrum DGU^®^, ATZ) is the participation in the “AltersTraumaRegister DGU©” mandatory.

There is a standardized questionnaire for entry in the register, with which the most important preoperative data are requested. In addition, surgical and postoperative data, as well as follow-up data, are collected via this questionnaire.

The infrastructure for documentation, data management, and data analysis is provided by AUC—Academy for Trauma Surgery (AUC—Akademie der Unfallchirurgie GmbH, Munich, Germany), a company affiliated to the German Trauma Society.

Data files from 2016 to 2020 were analyzed. The data from the “AltersTraumaRegister DGU©” received full approval from the Ethics Committee of the medical faculty of the Philipps University of Marburg, Germany, in 5 April 2018 (AZ 46/16).

Patients undergoing treatment for periprosthetic und pathological fracture were excluded, also patients from non-German hospitals.

For the analysis, the patients were divided into seven cohorts, depending on the time of surgery.

The seven cohorts were divided into weekdays and weekends. In order to be able to differentiate further, the cohorts were then divided into day and night and also into regular working hours:

The first cohort comprises all patients who underwent surgery during normal working hours from Monday to Friday between 07:00 and 15:30;
Cohort 2 comprises all patients who underwent surgery during working hours on weekdays between 15:31 and 19:59;
Cohort 3 comprises all patients who underwent surgery on Saturdays, Sundays, and public holidays between 07:00 and 15:30;
Cohort 4 comprises all patients who underwent surgery on weekends and on public holidays between 15:31 and 19:59;
Cohort 5 includes all patients who underwent surgery on Saturdays, Sundays, and public holidays between 20:00 and 23:59;
The sixth cohort includes all patients who underwent surgery between 00:00 and 7:00; and the seventh cohort includes all patients who underwent surgery during the week between 20:00 and 23:59.

The following pre-operative parameters were collected: time of admission, age and sex of patients, living situation, need for care, American Society of Anesthesiologists (ASA) classification on admission, anticoagulation, ability to walk before fracture, and fracture type.

The following data related to operations were used for the evaluation: time of surgery, mortality, time and place of discharge, type of anesthesia, ability to walk on the seventh post-operative day, surgical revisions, and method of surgery.

Follow-up data were determined by the following items: Re-admission, ability to walk after 120 days, change in ability to walk after 120 days, living situation after 120 days and change in this, revision surgery, and mortality.

The follow-up data is collected on a voluntary basis. Accordingly, there is a certain loss of data and a selection bias cannot be ruled out Although the follow-up data was evaluated in this study, the focus was on the in-hospital outcomes.

Also the timing of care- with regard to the regular working hours- according to the the Care Levels from the Data TraumaNetzwerk DGU^®^ (TNW) project. is collected. This data was provided from the AUC as well- as it is not an original part of the data collection of the geriatric trauma registry. The main idea of the TraumaNetzwerk DGU^®^ is to ensure the provision of comprehensive care through establishing regional trauma networks in Germany. Three levels of care have been defined for the clinics in the network, which include specific structural and process characteristics and key figures, as follows.

Local Trauma Centers (LTZs) play an essential role in the comprehensive treatment of common mono-injuries. They serve as initial points of contact for the care of seriously injured patients, with the important task of providing adequate initial treatment and targeted referral.

Regional Trauma Centers (RTZs) have the task of providing comprehensive emergency and definitive care for injured persons while maintaining and intensive care capacities.

Supra-regional trauma centers (SRTZs) have specific tasks and obligations for the comprehensive treatment of all multiple and severely injured persons, especially those with exceptionally complex or rare injury patterns [9].

This present publication complies with the publication guidelines of the ATR-DGU and is registered under the ATR-DGU project ID 2021-003.

All calculations were performed using the statistical software R v. 4.0.2 (Foundation for Statistical Computing, Vienna, Austria). For descriptive analyses, categorical data are presented as counts and percentages and continuous variables as mean with standard deviation (SD) or median with interquartile range (IQR) in the case of a skewed distribution. Statistical comparisons were made using the Χ^2^ test for categorical variables and one-way analysis of variance (ANOVA) or the Kruskal–Wallis test for continuous variables. Differences were considered statistically significant when *p* < 0.05. The null hypothesis to be examined was: There is no difference between patients following hip-related fracture while surgery took place during regular working hours and patients who underwent surgery outside of regular working hours.

## 3. Results

A total of 29,470 patients were included in the study from 108 hospitals. A total of 74% of patients underwent surgery within 24 h. From Table 1, it can be seen that the first cohort included 13,597 (46%), cohort 2 included 4853 (16%), cohort 3 included 5464 (19%), cohort 4 included 1895 (6%), cohort 5 included 848 (3%), cohort 6 included 527 (2%), and cohort 7 included 2286 (8%) patients.

### 3.1. Baseline Characteristics

Table 2 presents the collected baseline characteristics of the study population.

### 3.2. Age

The average age of all patients was 84.4 years.

**Figure 1:**
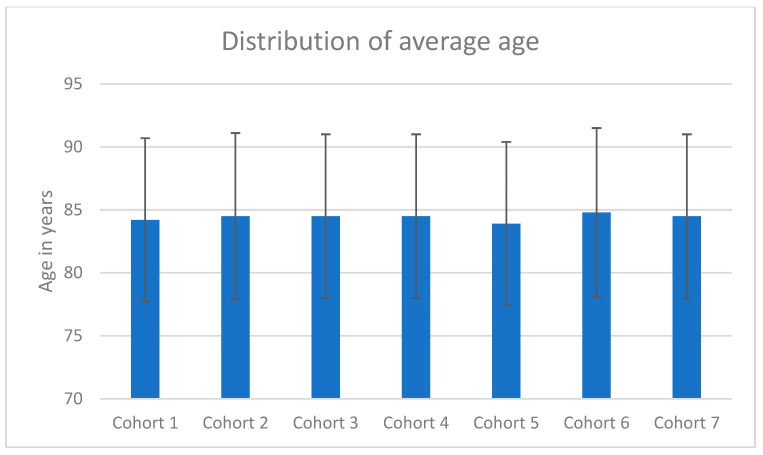
Distribution of average age.

In the first cohort, the average age was lowest at 84.2 years with a standard deviation of 6.5 years) while, in the sixth cohort, the average age was highest, at 84.8 years with a standard deviation of 6.7 years was shown in Figure 1.

When comparing the cohorts, the variance analysis revealed a *p*-value of 0.002 and, thus, a statistically significant difference between the groups.

### 3.3. ASA Classification

The majority of all patients were assigned to ASA III. The distribution of patients in the ASA classification was as follows: ASA I includes 350 (1.2%) patients, ASA II includes 6513 (22.5%) patients, ASA III includes 19,924 (68.7%) patients, ASA IV includes 2189 (7.5%) patients, and ASA V includes 27 (0.1%) patients.

**Figure 2:**
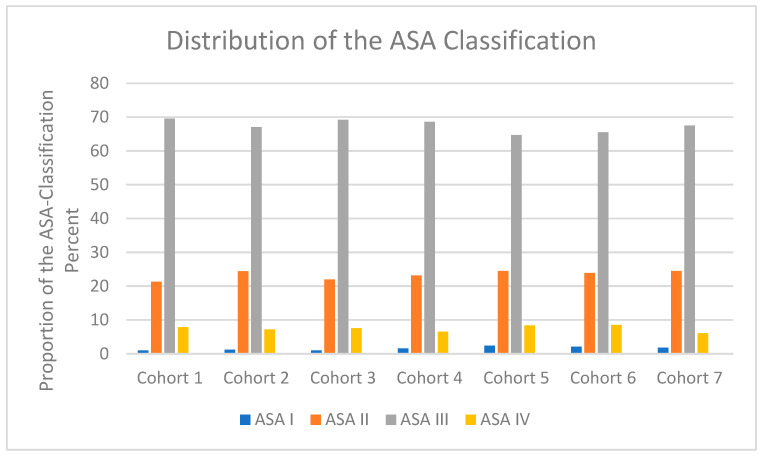
Distribution of the ASA classification.

Figure 2 shows the distribution of ASA classification in the different cohorts.

The calculated *p*-value in the chi-square test was less than 0.0001.

### 3.4. Timing of Care Between the Care Levels

**Figure 3:**
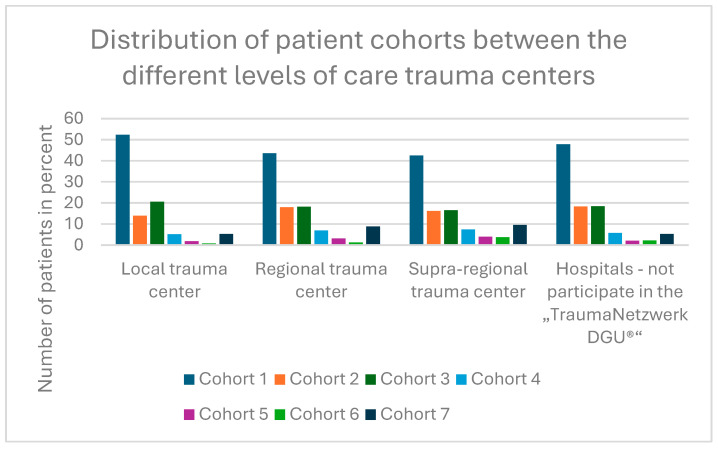
Distribution of the patient cohorts between the different levels of trauma centers.

The distribution of the time of surgery differs significantly between the care levels (*p* < 0.001). In supra-regional trauma centers, fewer patients underwent surgery during regular working hours, while more were treated late in the evening and at night as shown in Figure 3.

### 3.5. Distribution of Time to Surgical Treatment

A total of 21,854 (74.4%) patients underwent surgery within 24 h of admission to hospital.

The difference between the cohorts was statistically significant, as the calculated *p*-value was less than 0.001.

**Figure 4:**
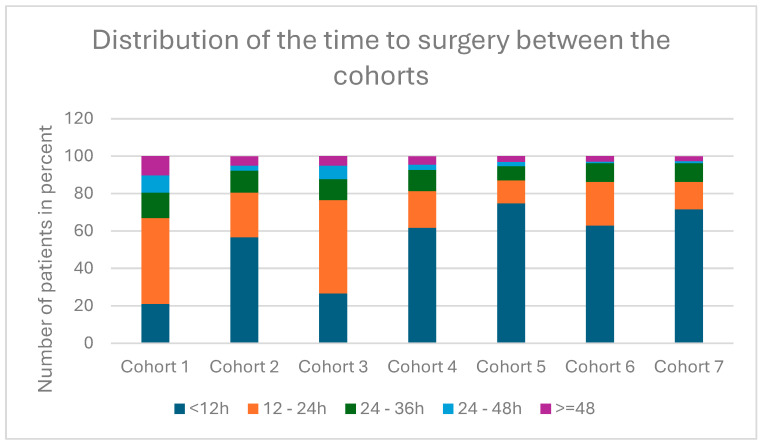
Distribution of time to surgical treatment between the cohorts.

As shown in Figure 4, patients in cohort 1 waited the longest for surgical treatment with a median time to surgery of 20.2 h after admission, with an interquartile range (IQR) of 13.8–27.6.

A statistically significant difference was also found here, with the *p*-value calculated in the Kruskal–Wallis test being less than 0.001.

### 3.6. Distribution of Time to Surgical Treatment at Different Levels of Care

**Figure 5:**
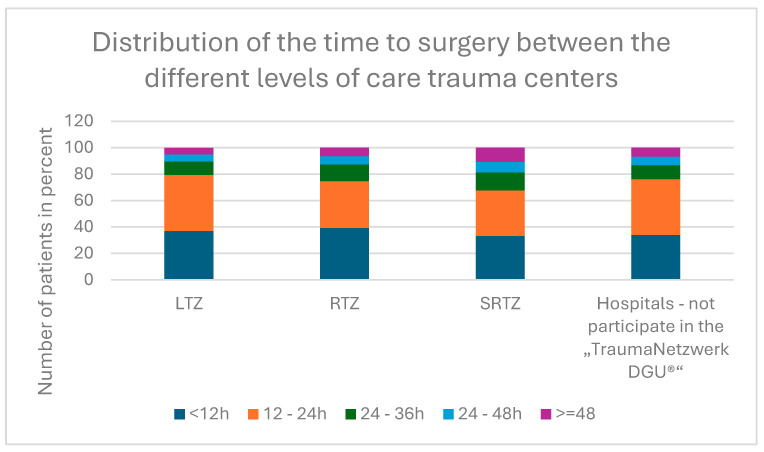
Distribution of time to surgical treatment between the different care levels.

Figure 5 shows that 7710 patients with proximal femur fractures had surgical treatment in local trauma centers, of which 6120 (79.3%) were treated within 24 h. Patients waited a median of 16.6 h and an IQR of 6.7–22.7.

A total of 10,204 patients with proximal femur fractures underwent surgery in regional trauma centers, of which 7620 (74.7%) were treated within 24 h. Surgical treatment was performed after a median of 16.3 h and an IQR of 6.5–24.

A total of 6564 patients with proximal femur fractures had their operation in supra-regional trauma centers, of which 4445 (67.7%) were treated within 24 h. Surgical treatment was performed after a median of 18.7 h and an IQR of 8.5–27.7.

In clinics not participating in the trauma network, a total of 2528 patients with proximal femur fractures underwent surgery, of which 1924 (76.1%) were treated within 24 h.

Surgical treatment was performed after a median of 17.9 h and an IQR of 7.4–23.7.

### 3.7. Fracture Entities

In total, there were 13,516 (45.9%) femoral neck fractures, 14,265 (48.5%) pertrochanteric fractures, 1134 (3.9%) subtrochanteric fractures, and 511 (1.7%) other proximal femoral fractures.

When comparing the fracture types between the cohorts, a *p*-value of less than 0.001 was calculated, meaning that there was a statistically significant difference between the cohorts with regard to the localization of the fracture.

In cohort 1, more than half of all surgically treated fractures were femoral neck fractures, a total of 7441 (54.8%).

In comparison, femoral neck fractures were treated least frequently in cohort 6. A total of 98 (18.6%) femoral neck fractures were registered and a further 13,106 pertrochanteric fractures were registered.

In comparison, pertrochanteric fractures were treated most frequently in the sixth cohort, 381 (72.3%). Meanwhile, in cohort 5, with only 527 (62.2%) pertrochanteric fractures, they were the most common.

Subtrochanteric fractures were rare in all cohorts. In percentage terms, most subtrochanteric fractures were treated in cohort 6.

### 3.8. Surgical Procedures

**Figure 6:**
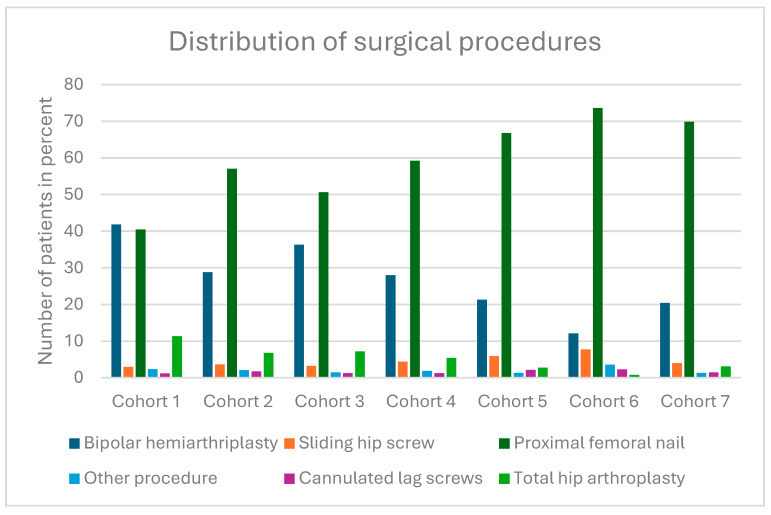
Distribution of surgical procedures between the cohorts.

A total of 29,691 surgical procedures were documented. The distribution of the different surgical procedures in the various cohorts is shown in Figure 6.

Hemiarthroplasty and total hip replacement were predominantly performed during normal working hours (Monday to Friday between 07:00 and 15:30). This means that 7293 (56%) of all arthroplasty operations were carried out during normal working hours, while only 6114 (37%) of all osteosynthesis operations were carried out during normal working hours. A total of 10,389 (35%) proximal femur fractures were treated with the implantation of bipolar hemiarthroplasty. Most bipolar hemiarthroplasties were performed in cohort 1, with the fewest in cohort 6.

A total of 2480 (8.4%) proximal femoral fractures were treated with total hip arthroplasty. In comparison, total hip arthroplasty was performed most frequently in cohort 1.

Furthermore, 1026 (3.5%) proximal femur fractures were treated with a dynamic hip screw, 394 (1.3%) proximal femur fractures were treated with cannulated lag screws, and 14,810 (50.3%) proximal femur fractures were with an intramedullary nail.

This type of osteosynthesis was also the most performed in cohort 6.

In addition, 592 (2%) surgical procedures were not described in detail.

### 3.9. Anticoagulation

Sicker patients with ASA ≥ 3 and patients on therapeutic anticoagulation were operated on significantly more often during standard working hours (*p* < 0.001).

**Figure 7:**
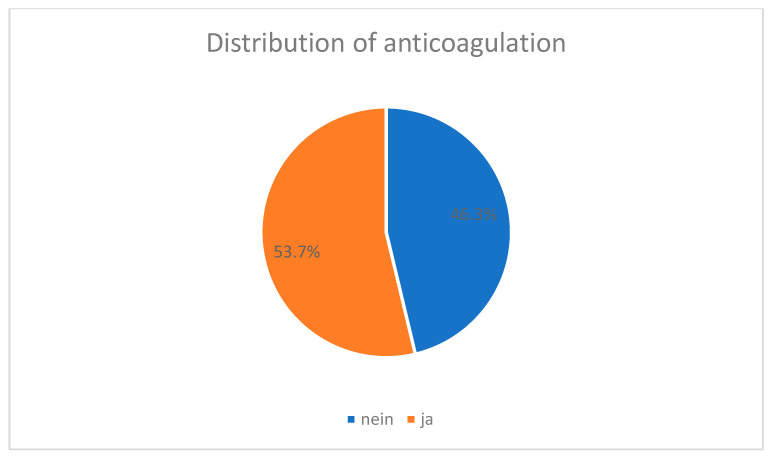
Distribution of anticoagulation (ja—yes; nein—no).

More than half of the patients were taking blood-thinning medication on admission. A total of 15,366 (53.7%) patients received some type of anticoagulation on admission, while 13,229 (46.3%) patients received no anticoagulation on admission which is shown in Figure 7.

The distribution in the different cohorts was as follows:

The proportion of patients receiving anticoagulation was highest in cohort 1. In cohort one, 7364 (55.8%) received anticoagulation and 5826 (44.2%) did not receive anticoagulation.

In cohort 2, 2492 (52.8%) received anticoagulation and 2231 (47.2%) did not receive anticoagulation.

In cohort 3, 2844 (53.6%) received anticoagulation and 2464 (46.4%) did not receive anticoagulation.

In cohort 4, 956 (51.8%) received anticoagulation and 890 (48.2%) did not receive anticoagulation.

In cohort 5, 387 (47.1%) received anticoagulation and 434 (52.9%) no anticoagulation. The proportion of anticoagulated patients was lowest here.

In cohort 6, 270 (53.7%) received anticoagulation and 233 (46.3%) did not receive anticoagulation.

In the seventh cohort, 1053 (47.8%) received anticoagulation and 1151 (52.2%) did not receive anticoagulation.

A comparison of the different cohorts revealed a *p*-value of less than 0.001 and, thus, a statistically significant difference.

### 3.10. Revision Rate and Mortality

A total of 1582 (5.4%) patients died in the first seven post-operative days after suffering a proximal femoral fracture: 773 patients (5.7%) died in Cohort 1, 259 (5.3%) in Cohort 2, 294 (5.4%) in Cohort 3, 87 (4.6%) in Cohort 4, 32 (5.1%) in Cohort 5, the lowest mortality was seen in Cohort 6 with 20 (3.8%) deaths, and 106 patients (4.6%) died in Cohort 7.

The difference between the cohorts was not statistically significant, with a *p*-value of 0.085.

In 1007 (3.4%) patients, a revision had to be performed due to complications during the initial stay. In cohort 1, 478 (3.5%) revisions were performed; in cohort 2, 156 (3.2%); in cohort 3, 165 (3.0%); in cohort 4, 76 (4.0%); in cohort 5, 25 (3.0%); in cohort 6, 21 (4.0%); and, in cohort 7, 86 (3.8%) underwent revision surgery.

No statistically significant difference was found between the cohorts, with a calculated *p*-value of 0.278.

The revision rate did not differ significantly across all groups, nor did mortality or morbidity.

Table 3 summarizes the most important results at a glance.

## 4. Discussion

The aim of this study was to determine the treatment reality in relation to the time of surgical treatment—namely, during working hours or outside of working hours—of hip-related fracture in Germany, and to investigate whether the timing of surgery has an influence on the outcomes of geriatric hip fracture patients.

Most of the patients were treated in regional trauma centers that are part of the TraumaNetzwerk DGU^®^. The 527 patients who had surgical treatment at night were distributed across only 61 clinics. Comparing the trauma levels according to the TraumaNetzwerk DGU^®^, it was found that supra-regional trauma centers perform surgeries at night more often than local trauma centers. This may be due to the fact that supra-regional trauma centers have the resources to care for geriatric trauma patients around the clock; On the other hand, it must also be taken into account that such hospitals often have to deal with much more serious injuries in the daily program and during off-peak working hours, so that the priority of pertrochanteric fracture here may not be the same as in smaller hospitals. Communication channels and agreements also often function more smoothly in leaner structures. However, compared to local trauma centers, patients waited statistically significantly longer for their care. There are certenly as mentioned above many reasons for this, but they can mainly be divided into patient-specific problems and logistical problems.

One could speculate that the sicker patients tend to be referred to larger, interdisciplinary hospitals, such as supra-regional trauma centers—however, the data available in the present evaluation is not sufficient to prove this—experience shows that the initial contact is often triggered regionally and not by the severity of the patient’s illness. The number of secondary transfers was not evaluated.

In general, multi-morbid patients wait longer for their surgical treatment. It can be assumed that these patients need to be optimized for surgical care for longer than 24 h pre-operatively, as has been documented in several international studies [10,11,12,13,14]. Another explanation could be the use of anticoagulants; however, this has been refuted in the study of Gleich et al. [15]. Logistical reasons for the later treatment of proximal femoral fractures are a lack of operating capacity or a lack of expertise due to parallel emergency care. These reasons can be found in several European publications [11,14]. The lack of resources in the regular service and early service business also explains care during the late evening or night.

In our dataset, 45% of the fractures were femoral neck fractures and 55% were fractures in the trochanteric region.

Femoral neck fractures were statistically significantly more frequently treated surgically during normal working hours. This shows that hemi- and total-hip arthroplasties were, by far, the most frequently implanted during normal working hours. The treatment of pertrochanteric fractures and osteosynthesis for femoral neck fractures were accordingly performed significantly more frequently at night and in the late evening. These differences can be explained by the available resources and expertise in the hospital. Another reason could be the different personnel requirements for these two types of care. As a rule, three or at least two surgeons are required to perform an arthroplasty, while a femoral nail can be implanted by a single person. An important reason for the increased provision of total hip arthroplasties in the morning hours is that bipolar hemiarthroplasties were important for the number of operations performed by the main surgeons for recording in the arthroplasty register until 2020.

A similar trend can be seen in the study of Forssten, using registry data from Sweden [16]. Data from Canada, as reported by Pincus et al., only showed a significant difference between care during regular working hours and care on duty in the case of total arthroplasty. The researchers from Canada were able to determine that treatment at night took longer and was typically performed by a less-experienced surgeon [17].

In this study, almost three-quarters of all patients received surgical treatment within 24 h. In the rest of Europe, treatment within 24 h is much less common.

There are very little data available for the rest of Europe regarding when hip fractures are operated on, and the operation rate during working hours differs greatly between the studies. The registry study by Forssten et al. showed that, in Sweden, only just over a third of hip fractures underwent surgery during working hours [16], weather the studies by Chacko et al. and Barinaga et al. from America reported a rate of around 30% for on-duty operations [18,19]. In Canada, on the other hand, two-thirds of patients underwent surgery during office hours [20]; in our study, more than half of the patients underwent surgery during office hours. In China, meta-analyses have been performed to demonstrate the safety of on-duty hours, as otherwise timely treatment of hip fractures does not appear to be possible [21,22].

The indication for nocturnal surgery must be carefully considered, even if there are studies from Registry for Geriatric Trauma (ATR-DGU) that also describe treatment between 24 and 48 h as safe [7].

Nevertheless, care at night appears to be a disproportionate burden not only for the patient, but also for all those involved in patient care.

When analyzing the anticoagulation, as a further possible reason for delayed surgery, it was noticeable that more than half of the patients were treated with at least one type of medication that influences the coagulation system. Just over a fifth of all patients were taking therapeutic anticoagulants.

The proportion of anticoagulated patients was highest among patients who underwent surgery during normal working hours. The differences were statistically significant.

Kolodychuk et al. investigated the influence of direct oral anticoagulants on peri-operative complications after osteosynthesis and arthroplasty following proximal femur fractures. They found that there was no increase in complications, even if the operation was performed within 24 h of the last dose. Therefore, they recommended prompt treatment, even if direct oral anticoagulation is taken [23]. This is in line with the recommendations of Schermann et al. [24]. For treatment with vitamin K antagonists, antagonization with vitamin K and PPSB and supply within 24 h also appears to be safe [23,24,25,26]. Patients receiving anticoagulation should, therefore, generally not wait longer than 24 h for their operation. It seems sensible to operate on these patients the day after the accident, during normal working hours.

A good 3% of patients had to be revised during their initial stay. There was no significant difference between the cohorts. There was also no significant difference in a European comparison [27]. It is interesting to note that, despite the significant differences in fracture entity and surgical treatment, the revision rate was very similar across all cohorts.

The complications can be attributed to patient-specific characteristics, such as general condition, osteoporosis, fracture type, and mental status, as well as to surgical characteristics such as reduction, osteosynthesis, soft tissue injury, and implant choice [28,29].

A total of 5% of patients died during the initial hospitalization. There was no statistically relevant difference between the cohorts.

In a European comparison, the mortality rate in the available registry data was also estimated at 4–5% [27].

The length of stay of patients who underwent surgery at night was statistically significantly shorter in comparison. However, the reason for this is most likely not the better outcome of these patients, but the fact that they were admitted at night after having already received treatment.

One reason for the generally somewhat longer length of stay for patients treated during normal working hours may also be the higher proportion of arthroplasty procedures performed during regular working hours [30]. In our study, as already reported elsewhere, transfer for further inpatient treatment does not appear to have caused an increase in the length of stay [31].

In a European comparison, patients in Germany received the longest inpatient treatment in acute care clinics [27]. This is certainly a side effect of the German DRG system, which defines a regular length of stay for respective interventions, on the basis of which the revenues are calculated.

In order to be able to treat these fractures during normal working hours, logistical and personnel requirements must be met. Treatment during normal working hours within 24 h creates the best possible personnel situation. In the future, an operating room for emergencies such as proximal femur fractures would be a good option to be able to treat emergencies promptly during the day.

Also the ability to concentrate and speed decrease with increasing fatigue or during on-call times [32,33]. For example, in the field of lung transplantation, a subgroup analysis revealed significant correlations with 90-day mortality. In particular, lower mortality rates were observed for donor clamp times between 8 am and 1 pm [34].

In a study from the Netherlands, the weekend effect in proximal femur fractures was retrospectively investigated. The study compared 1257 patients who were admitted on weekdays with 546 patients who were admitted at the weekend or on public holidays. Patients were statistically significantly more likely to undergo surgery within 24 h on weekends; mortality after 30 days and one year did not differ significantly [35].

However, there are also studies that provide the opposite results.

Foss and Kehlet followed up patients with proximal femoral fractures who were hospitalized during the weekend or holidays. In their prospective study, they were able to show that the mortality of patients admitted at the weekend or on public holidays was statistically significantly higher five and 30 days postoperatively than that of patients admitted during the week [36].

Bhattachary and colleagues from Boston, similar to the study by Chacko et al. [19], compared patients who were operated on during the day between 7:00 am and 5:00 pm and patients who were operated on between 5:00 pm and 7:00 am. In the meantime, a special trauma operating room was established in Boston so that Bhattachary could also compare the outcome of patients operated on after the introduction of this extra room with the day and duty cohorts. When analyzing the data, it was found that hip fracture surgeries and femoral nailings done at night were noted to have a higher incidence of surgical complications (*p* < 0.04 and *p* < 0.036) [37].

### Limitations of the Study

The findings of the present study are limited by several factors. First of all, this is a retrospective study with all known potential biases, like selection bias and confounding. Moreover, data is strongly dependent on documentation quality. Also not all data were collected from every single patient. Unfortunately, this is commonly the case for registry data. Nevertheless, the strengths of this study are the high number of participants, unifying the present study- population and reducing the multiple confounding factors that complicate the evaluation of such treatment data. Patients from multiple centers all over Germany were included in this study, and all patients were treated in an ortho-geriatric setting.

## 5. Conclusions

Although we manage to treat the majority of patients in Germany within 24 h, only 46% of patients are operated on within standard working hours. In terms of the available outcome parameters, this does not appear to result in any significant disadvantage for patients. The link between pre-operative length of stay and time of surgery suggests that the focus here is not on emergency fracture treatment but, rather, organizational reasons.

Nevertheless, night work and fatigue affect concentration and post-operative results in many areas, as we know. Consequently, patient care during normal working hours within 24 h creates the best possible initial situation for the patient, as significantly more personnel resources are available during normal working hours. The aim should be to provide the logistical and personnel requirements for this.

Further, as performed in some hospitals, an geriatric trauma emergency operating room can be a possible option, on a day-to-day basis, such that patients with proximal femur fractures and other emergencies can undergo surgery promptly during the day.

In addition to the best possible patient care, there could also be a benefit for all those working in emergency care, as resources could be conserved during working hours and the workload on duty could be reduced.

## Figures and Tables

**Table 1:** Distribution of patients between the cohorts.

Cohort	Time of Surgery	Total of Patients
Cohort 1	07:00–15:30/regular working hours	13,597 (46%)
Cohort 2	15:31–19:59/weekdays	4853 (16%)
Cohort 3	07:00–15:30/weekends and public holidays	5464 (19%)
Cohort 4	15:31–19:59/weekends and public holidays	1895 (6%)
Cohort 5	20:00–23:59/weekends and public holidays	848 (3%)
Cohort 6	0:00–6:59	527 (2%)
Cohort 7	20:00–23:59/weekdays and nights	2286 (8%)

**Table 2:** Baseline characteristics.

	All Patients
Average Age (Mean)	84.4 years (SD 6.5)
Gender Distribution	21,185 (72%) female 8234 (28%) male
ASA Classification	ASA I—350 patients (1.2%) ASA II—6513 patients (22.5%) ASA III—19,924 patients (68.7%) ASA IV—2189 patients (7.5%)
Distribution of the patients between the different levels of trauma centers	Local trauma center—7723 patients Regional trauma center—10,235 patients Supra-regional trauma center—6580 patients Other Hospitals—2539 patients
Fracture entities	13,516 (45.9%) femoral neck fractures 14,265 (48.5%) pertrochanteric fractures 1134 (3.9%) subtrochanteric fractures 511 (1.7%) not further classified
Length of stay (Mean)	17.2 days (SD 8.7)
Anticoagulation	15,366 (53.7%) some type of anticoagulation on admission 13,229 (46.3%) no anticoagulation on admission
Surgical Revision	1007 (3.4%)

**Table 3:** Summarized Results.

Time to surgery	
Total patients	29,470
Surgery < 24h	21,854 (74%)
Surgery > 24h	7539 (26%)
Distribution of the operation during working hours (Monday to Friday between 07:00 and 15:30)	
Osteosynthesis	6114 (37%)
Arthroplasties	7293 (56%)
Time to surgery between the different levels of care trauma centers Surgery < 24h	Local trauma centers: 6120 (79.3%) Regional trauma centers: 7620 (74.7%) Supra-regional trauma centers: 4445 (67.7%) Others: 1924 (76.1%)
Revision rate, morbidity and mortality between the cohorts	No significance

## Data Availability

Restrictions apply to the availability of these data. Data were obtained from the Registry for Geriatric Trauma (ATR-DGU) and are available from the Academy for Trauma Surgery (AUC) with the permission of the Working Committee on Geriatric Trauma Registry (AK ATR) of the German Trauma Society (DGU).
